# Depleting Yes‐Associated Protein in *Gli1*‐Expressing Cells Attenuates Peritoneal Dialysis‐Induced Peritoneal Fibrosis

**DOI:** 10.1111/jcmm.70516

**Published:** 2025-03-25

**Authors:** Chia‐Lin Wu, Jhih‐Wen Hsu, Ya‐Chi Chan, Jenn‐Yah Yu, Yi‐Liang Tsai, Der‐Cherng Tarng

**Affiliations:** ^1^ Department of Post‐Baccalaureate Medicine, College of Medicine National Chung Hsing University Taichung Taiwan; ^2^ School of Medicine Chung Shan Medical University Taichung Taiwan; ^3^ Division of Nephrology, Department of Internal Medicine Changhua Christian Hospital Changhua Taiwan; ^4^ Renal Medicine Laboratory Changhua Christian Hospital Changhua Taiwan; ^5^ National Yang Ming Chiao Tung University Taipei Taiwan; ^6^ Department and Institute of Physiology National Yang Ming Chiao Tung University Taipei Taiwan; ^7^ Division of Nephrology, Department of Medicine Taipei Veterans General Hospital Taipei Taiwan

**Keywords:** *Gli1*, myofibroblast, peritoneal fibrosis, yes‐associated protein

## Abstract

Long‐term peritoneal dialysis (PD) leads to peritoneal damage and chronic inflammation, resulting in peritoneal fibrosis (PF). Emerging evidence suggests that yes‐associated protein (YAP) is a key player in fibrogenesis across various organs. However, its role in PD‐induced PF remains unclear. We used NIH/3T3 cells, primary mouse fibroblasts, and conditional YAP knockout (CKO) mice with glioma‐associated oncogene 1 (*Gli1*)‐specific YAP deletion. The effects of YAP knockdown and verteporfin, a YAP inhibitor, on fibroblast‐to‐mesenchymal transition (FMT) and angiogenesis were evaluated. Transforming growth factor‐beta (TGF‐β) induced YAP expression and promoted fibroblast‐to‐myofibroblast transition (FMT) in 3T3 fibroblasts, upregulating collagen 1A1, α‐smooth muscle actin (α‐SMA), and connective tissue growth factor (CTGF). YAP knockdown and verteporfin treatment reduced these FMT markers and inhibited smad2/3 phosphorylation. In vivo, YAP and *Gli1*‐expressing cells were upregulated in PD‐induced PF. Conditional YAP knockout in *Gli1* ^+^ cells and verteporfin treatment significantly reduced fibrosis and α‐SMA, collagen 1, TGF‐β, CTGF, and phosphorylated smad2/3 expression in the peritoneum and peritoneal angiogenesis. YAP plays a pivotal role in FMT during PD‐induced PF. Conditional YAP deletion in *Gli1*‐expressing cells and verteporfin treatment represent promising antifibrotic strategies for long‐term PD patients.

## 1 Introduction

Peritoneal dialysis (PD) is a vital treatment option for end‐stage renal disease (ESRD). PD relieves uremic symptoms, reduces complications, improves quality of life, and prolongs the survival of ESRD patients. Compared with haemodialysis, PD is more autonomous and flexible in terms of treatment and patient satisfaction and has a lower cost [1, 2]. However, current peritoneal dialysis fluids (PDFs), which contain high glucose concentrations, glucose degradation products, and acidity, are bioincompatible [3]. Long‐term use of these bioincompatible PDFs can damage the peritoneum, induce chronic inflammation, and lead to peritoneal fibrosis and neovascularization, followed by ultrafiltration failure and inadequate clearance of uremic toxins [4]. Moreover, encapsulating peritoneal sclerosis (EPS) may develop and lead to malnutrition and a high mortality rate in some patients undergoing long‐term PD [5]. These shortcomings have primarily limited the use of PD and need to be solved.

The Hippo signalling pathway is conserved in mammals. The Hippo pathway has been shown to regulate cell proliferation, organ development, and tumorigenesis [6, 7]. Yes‐associated protein (YAP) and transcriptional coactivator with PDZ‐binding motif (TAZ) are important downstream effectors of the Hippo signalling pathway [8]. Our previous study reported the protective effect of TAZ against renal ischaemia–reperfusion injury (IRI) [9]. Recently, studies have revealed that YAP can affect fibrogenesis in some organs [10, 11].

The glioma‐associated oncogene (*Gli*) pathway has also been shown to exert profibrotic effects in experimental models of fibrosis [12]. *Gli1* activation was observed in myofibroblasts, and *Gli1* ^+^ cell‐specific YAP/TAZ knockout by tamoxifen treatment was reported to ameliorate the development of renal fibrosis following unilateral ureteral obstruction [13]. Strippoli et al. suggested that YAP/TAZ could coordinate with caveolin‐1 to regulate fibrogenesis by modulating the mesothelial‐to‐mesenchymal transition [14]. However, the role of YAP and whether *Gli1*‐expressing cells in the peritoneum contribute to myofibroblast differentiation in peritoneal fibrosis has not been thoroughly investigated. Thus, we hypothesised that YAP expression in *Gli1*‐expressing cells could promote the transdifferentiation of fibroblasts into myofibroblasts and the development of fibrosis in the peritoneum. We performed this study to examine the profibrotic role of YAP in peritoneal fibrosis. Our results suggest that *Gli1* is activated in peritoneal myofibroblasts and that YAP knockout in *Gli1‐*expressing cells substantially suppresses fibrosis induced by PDF.

## 2 Methods

### 2.1 Cell Culture

NIH/3T3 (3T3) cells, which are a mouse fibroblast line, were purchased from the Bioresource Collection and Research Center (BCRC, Hsin‐Chu, Taiwan). According to the American Type Culture Collection, 3T3 cells were incubated in Dulbecco's modified Eagle's medium (DMEM, HyClone) supplemented with 10% calf bovine serum, 3.7 g/L sodium bicarbonate, and 100 U/mL penicillin/streptomycin at 37°C and 5% CO_2_. 3T3 cells (3 × 10 ^5^) were seeded in 6‐well culture plates and incubated overnight. The cells were cultured in serum‐free medium for 24 h and then treated with 1 or 10 ng/mL transforming growth factor beta (TGF‐β) (catalogue no. 763102, BioLegend) for 24 h. In vitro knockdown of YAP was performed using RNA interference. 3T3 cells were treated with YAP siRNA (1 μg, SASI Mm01_00022141/YAP1, Sigma; sequence: 5′‐CCAAUAGUUCCGAUCCCUU‐3′) for 24 h and treated with *TGF‐β for* 24 h. 3T3 cells were treated with 500 nM verteporfin (catalogue no. 17334, Cayman) for inhibition of YAP and treated with *TGF‐β for* 24 h. All cell culture experiments were independently repeated more than three times.

### 2.2 PD‐Induced Peritoneal Fibrosis Animal Model

The method for inducing peritoneal fibrosis was modified from a previous study [15]. Briefly, the mice were intraperitoneally injected with 2 mL of peritoneal dialysis fluid containing 4.25% glucose (Dianeal, Baxter Healthcare) supplemented with 40 mM methylglyoxal daily for 3 weeks. Control mice were intraperitoneally injected with the same volume of phosphate‐buffered saline daily for 3 weeks.

### 2.3 Conditional Knockout of YAP in Mice

Conditional *YAP* allele‐floxed mice were crossed with transgenic mice harbouring tamoxifen‐inducible CreERT2 driven by the *Gli1* promoter to generate conditional *YAP*‐knockout mice (*YAP* ^*f/f*^;*Gli1*‐CreERT2^+/−^, shown in Figure S1). Age‐matched (8–12 weeks of age) male littermates were used for all experiments. The mice were on the C57BL/6J background. Tamoxifen (75 mg/kg weight) (catalogue no. T5648, Sigma–Aldrich) was administered intraperitoneally for 5 consecutive days. Then, the mice were subjected to 21 days of peritoneal fibrosis induction after a 9‐day washout period for tamoxifen. *YAP* ^*f/f*^ mice were obtained from Professor Duojia Pan at the University of Texas Southwestern Medical Center (Dallas, Texas, USA), and *Gli1*‐CreERT2 mice were purchased from The Jackson Laboratory (Farmington, Connecticut, USA). Verteporfin (25 mg/kg weight) was administered intraperitoneally every other day for 21 days. The Institutional Animal Care and Use Committee of Changhua Christian Hospital (Changhua, Taiwan) approved all animal use protocols (approval no. CCH‐AE‐108‐014).

### 2.4 Immunofluorescence Analysis and Confocal Microscopy

The frozen peritoneal tissue was cut into 8 μm‐thick sections. The sections were rehydrated in PBS for 10 min, washed with PBS twice, and blocked with 10% goat serum for 30 min at room temperature (RT). The sections were incubated with the following primary antibodies at 4°C overnight: anti‐α‐SMA (1:1000; catalogue no. ab32575; Abcam), anti‐YAP (1:150; catalogue no. sc‐101199; Santa Cruz), anti‐Gli1 (1:100; catalogue no. AF3455; R&D), anti‐COL1A1 (1:1000; catalogue no. 72026; Cell Signalling Technology), anti‐TGF‐β (1:100; catalogue no. sc130348; Santa Cruz), anti‐connective tissue growth factor (CTGF; 1:200; catalogue no. sc101586; Santa Cruz), anti‐phosphorylated mothers against decapentaplegic homologue 2 (smad2; 1:50; catalogue no. 18338; Cell Signalling Technology), anti‐phosphorylated mothers against decapentaplegic homologue 3 (smad3; 1:75; catalogue no. ab52903; Abcam), and anti‐cluster of differentiation 31 (CD31; 1:100; catalogue no. ab182981; Abcam). After being washed with PBS three times, the sections were incubated with fluorescence‐conjugated secondary antibodies, including Alexa Fluor 488 anti‐rabbit (1:300; catalogue no. 111–545‐003; Jackson ImmunoResearch Inc.) and rhodamine red anti‐mouse (1:100; catalogue no. 115–295‐003; Jackson ImmunoResearch Inc.) and Alexa Fluor 488 donkey anti‐goat (1:300; catalogue no. ab150129; Abcam) for 1 h at RT. Finally, the cell nuclei were counterstained with DAPI. Peritoneal tissue coexpressing α‐SMA, YAP, and Gil1 was examined using a fluorescence microscope (DMi8; Leica) and a laser scanning confocal microscope (FV1200; Olympus). The fluorescence intensity in the peritoneum was quantified by ImageJ software (Media Cybernetics, Bethesda, Maryland, USA).

### 2.5 Immunohistochemistry

Peritoneal tissues were fixed in 10% paraformaldehyde. These tissues were cut into 5 μm sections following dehydration and paraffin embedding. Immunohistochemistry was performed using a microwave‐based antigen retrieval technique [16]. The tissue sections were stained with primary antibodies against COL1A1 (1:250; catalogue no. 72026, Cell Signalling). A Polink‐2 Plus HRP‐conjugated anti‐rabbit detection kit (GBI labs, Mukilteo, USA) was used to visualise the tan colour that indicated positive signals. Haematoxylin was used for counterstaining. The integrated optical density (IOD) of collagen 1A1 per low‐power field (LPF) was quantified by ImageJ software. At least 4 images at 200x magnification were taken from each mouse.

### 2.6 Staining for Peritoneal Fibrosis

Masson's trichrome staining was used to evaluate fibrosis in the peritoneum as previously described [17]. Briefly, peritoneal tissues were fixed in 10% paraformaldehyde. After dehydration and paraffin embedding, the tissues were cut into 5 μm sections. The peritoneal tissue sections were stained with a Masson's Trichrome Kit (ScyTek Laboratories, Logan, Utah, USA) according to the manufacturer's instructions to assess the severity of peritoneal fibrosis and collagen accumulation in the peritoneum. The peritoneal thickness was measured using ImageJ software.

### 2.7 Western Blotting

3T3 cells were lysed in RIPA buffer at 4°C for 30 min. The protein concentration of the lysate was quantified using a bicinchoninic acid assay kit (Thermo Scientific). We used a nuclear protein extraction kit (BRARZ106, TOOLS) according to the manufacturer's instructions. Immunoblotting was performed using primary antibodies against YAP (catalogue no. 14074; Cell Signalling), COL1A1 (catalogue no. 72026; Cell Signalling), α‐smooth muscle actin (catalogue no. 19245; Cell Signalling), CTGF (catalogue no. Ab209780; Abcam), Gli1 (catalogue no. Ab217326; Abcam), smad2/3 (catalogue no. 8685; Cell Signalling), phospho‐smad2 (catalogue no. 3108; Cell Signalling), phospho‐smad3 (catalogue no. 9520; Cell Signalling), Lamin A/C (catalogue no. Ab108922; Abcam), β‐actin (catalogue no. NB600‐501; Novus), and GAPDH (catalogue no. IR3‐8; iREAL). HRP‐conjugated anti‐rabbit (catalogue no. GTX213110‐01; GeneTex) and anti‐mouse (catalogue no. GTX213111‐01; GeneTex) secondary antibodies were used. For western blotting, β‐actin or GAPDH was used as an internal control.

### 2.8 Statistical Analysis

The data are expressed as the mean (standard error of the mean). Differences among the groups were compared by Student's *t* test or one‐way ANOVA. No criteria were used for including and excluding animals during the experiment and data points during the analysis. A two‐tailed *p* value < 0.05 was considered to indicate statistical significance. All the statistical analyses were performed using SPSS software (version 20, SAS Institute Inc., Cary, NC, USA) and GraphPad Prism (version 9.5; Stata Corp, College Station, TX, USA).

## 3 Results

### 3.1 TGF‐β Induces YAP Expression and the Fibroblast‐To‐Myofibroblast Transition (FMT)

**FIGURE 1:**
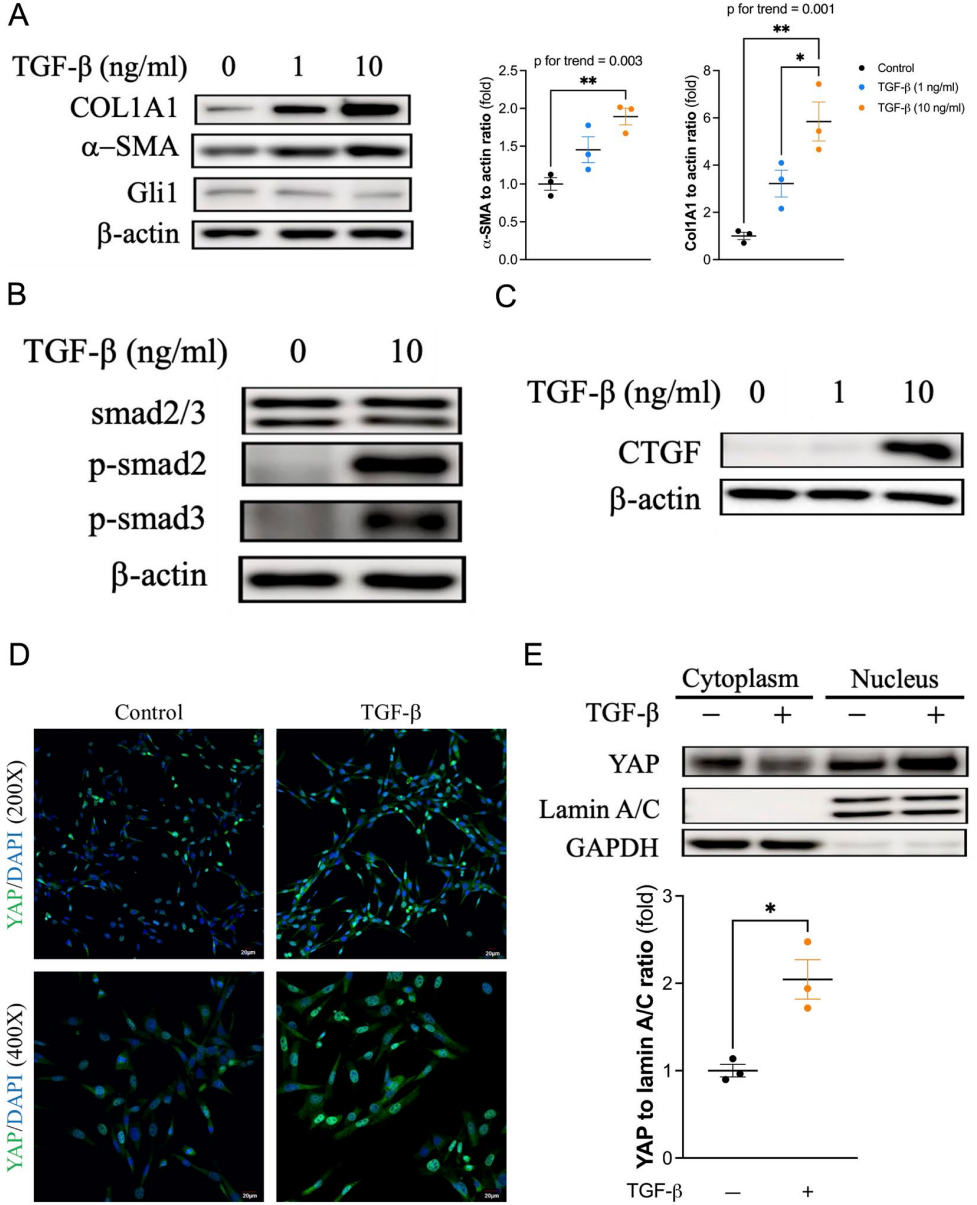
Yap signalling is activated during the fibroblast‐to‐myofibroblast transition (FMT). (A) 3T3 mouse fibroblasts were transformed to α‐SMA‐ and COL1A1‐expressing myofibroblasts by TGF‐β in a dose‐dependent manner. 3T3 fibroblasts also expressed *Gli1*. The left panel shows representative western blots and the right panel shows the results (mean ± SEM) of densitometric analysis of three independent protein extracts. (B) 3T3 cells were treated with 10 ng/mL TGF‐β for 30 min, and downstream phosphorylation of smad2 and smad3 was activated. (C) TGF‐β induced the expression of CTGF during FMT. (D) TGF‐β enhanced the expression and nuclear translocation of YAP. Confocal fluorescence microscopy showed increased YAP staining, which colocalized with DAPI in TGF‐β‐treated 3T3 fibroblasts. Scale bar, 20 μm. (E) YAP was increased in the nuclear fraction after treatment with 10 ng/mL TGF‐β for 2 h (*n* = 3 for each group). *p < 0.05, **p < 0.01. One‐way ANOVA followed by Tukey's post hoc test for (A), and unpaired Student's *t* test for (E). α‐SMA, alpha‐smooth muscle Actin; COL1A1, collagen 1A1; CTGF, connective tissue growth factor; DAPI, 4′, 6‐diamidino‐2‐phenylindole; GAPDH, glyceraldehyde‐3‐phosphate dehydrogenase; *Gli1*, glioma‐associated oncogene homologue 1; SEM, standard error of the mean; TGF‐β, transforming growth factor beta; YAP, yes‐associated protein.

3T3 mouse fibroblasts expressed *Gli1* (Figure 1A). In addition, TGF‐β significantly induced the expression of collagen 1A1 and α‐SMA in a dose‐dependent manner in 3T3 fibroblasts. Moreover, the phosphorylation of smad2 and smad3 and the expression of CTGF were activated during TGF‐β‐induced FMT (Figure 1B,C). In addition, YAP signalling was activated during FMT because nuclear translocation in 3T3 fibroblasts was enhanced by 10 ng/mL TGF‐β treatment (Figure 1D,E).

### 3.2 Suppressing YAP Inhibits FMT in Fibroblasts

**FIGURE 2:**
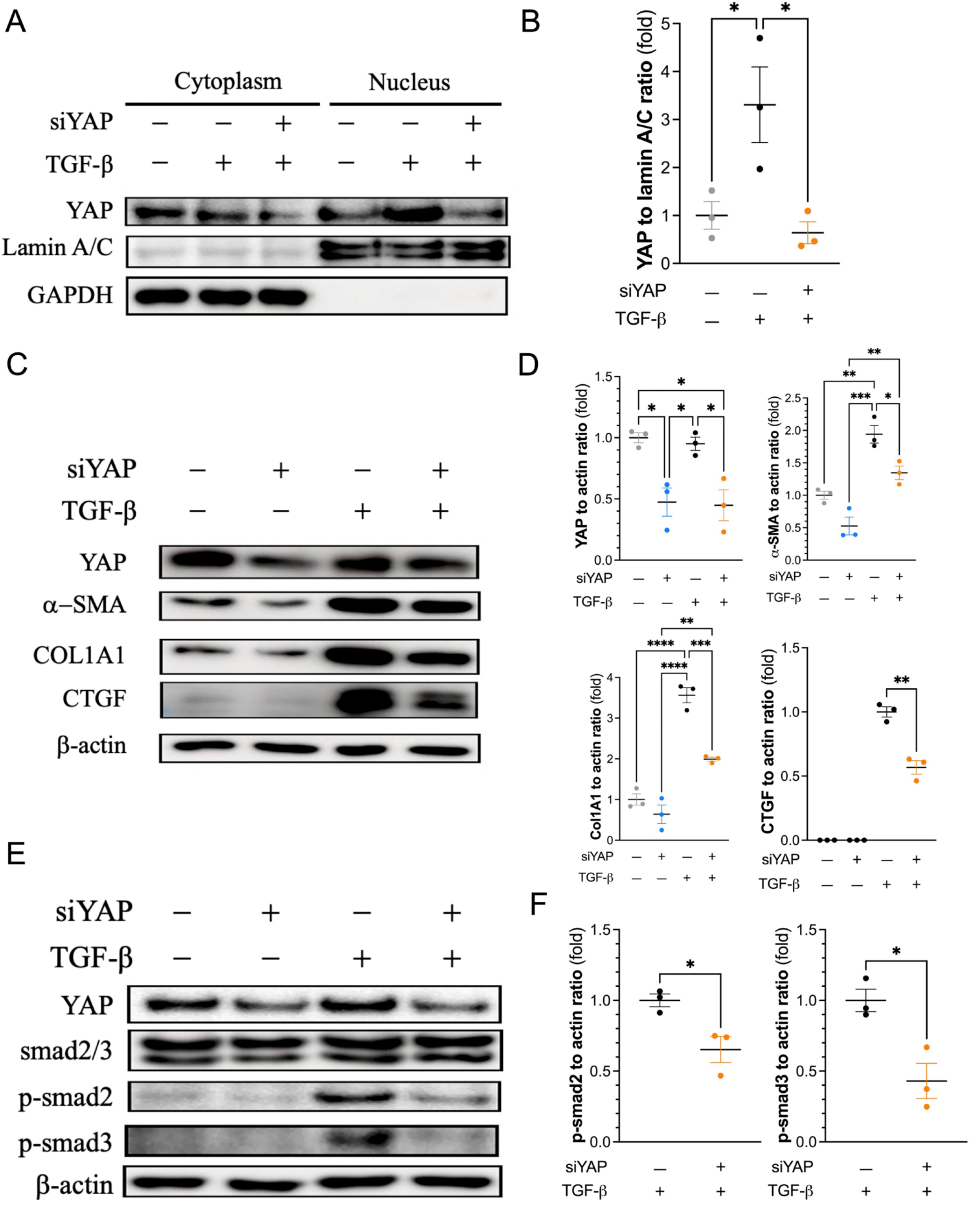
Yap knockdown inhibits fibroblast‐to‐myofibroblast transdifferentiation. (A) YAP siRNA reduced YAP expression in the cytoplasm and its nuclear translocation in 3T3 fibroblasts. Densitometry (data expressed as the mean ± SEM) showed that the YAP‐to‐lamin A/C ratio was significantly decreased in YAP siRNA‐ and TGF‐β‐treated 3T3 cells compared with those treated with TGF‐β alone (B). YAP siRNA also reduced YAP expression in whole cell lysates of 3T3 fibroblasts. YAP siRNA suppressed the TGF‐β‐induced expression of α‐SMA, collagen 1A1, and CTGF in 3T3 cells (C). The results of densitometric analyses (mean ± SEM) of three independent protein extracts (D). YAP siRNA attenuated the TGF‐β‐induced phosphorylation of smad2 and smad3 in 3T3 cells (E and F). *p < 0.05, **p < 0.01, ***p < 0.001, ****p < 0.0001. One‐way ANOVA followed by Tukey's post hoc test for (B and D), and unpaired Student's *t* test for (F). *n* = 3 for each group. α‐SMA, alpha‐smooth muscle Actin; COL1A1, collagen 1A1; CTGF, connective tissue growth factor; GAPDH, glyceraldehyde‐3‐phosphate dehydrogenase; SEM, standard error of the mean; Smad, mothers against decapentaplegic; TGF‐β, transforming growth factor beta; YAP, yes‐associated protein.

The increase in the nuclear translocation of YAP induced by TGF‐β was suppressed by siRNA in 3T3 cells (Figure 2A,B). In addition, knockdown of YAP inhibited the expression of YAP, α‐SMA, collagen 1A1, and CTGF, and these effects were augmented by TGF‐β (Figure 2C,D). The expression of α‐SMA and collagen 1A1 tended to be suppressed by YAP knockdown in fibroblasts without TGF‐β (Figure 2C,D). Moreover, TGF‐β triggered the downstream phosphorylation of smad2 and smad3, although their total levels were unchanged. YAP knockdown inhibited the phosphorylation of smad2 and smad3 (Figure 2E,F). Furthermore, knocking down YAP prohibited FMT in primary mouse fibroblasts (Figure S2A).

### 3.3 Verteporfin Inhibits YAP and FMT

**FIGURE 3:**
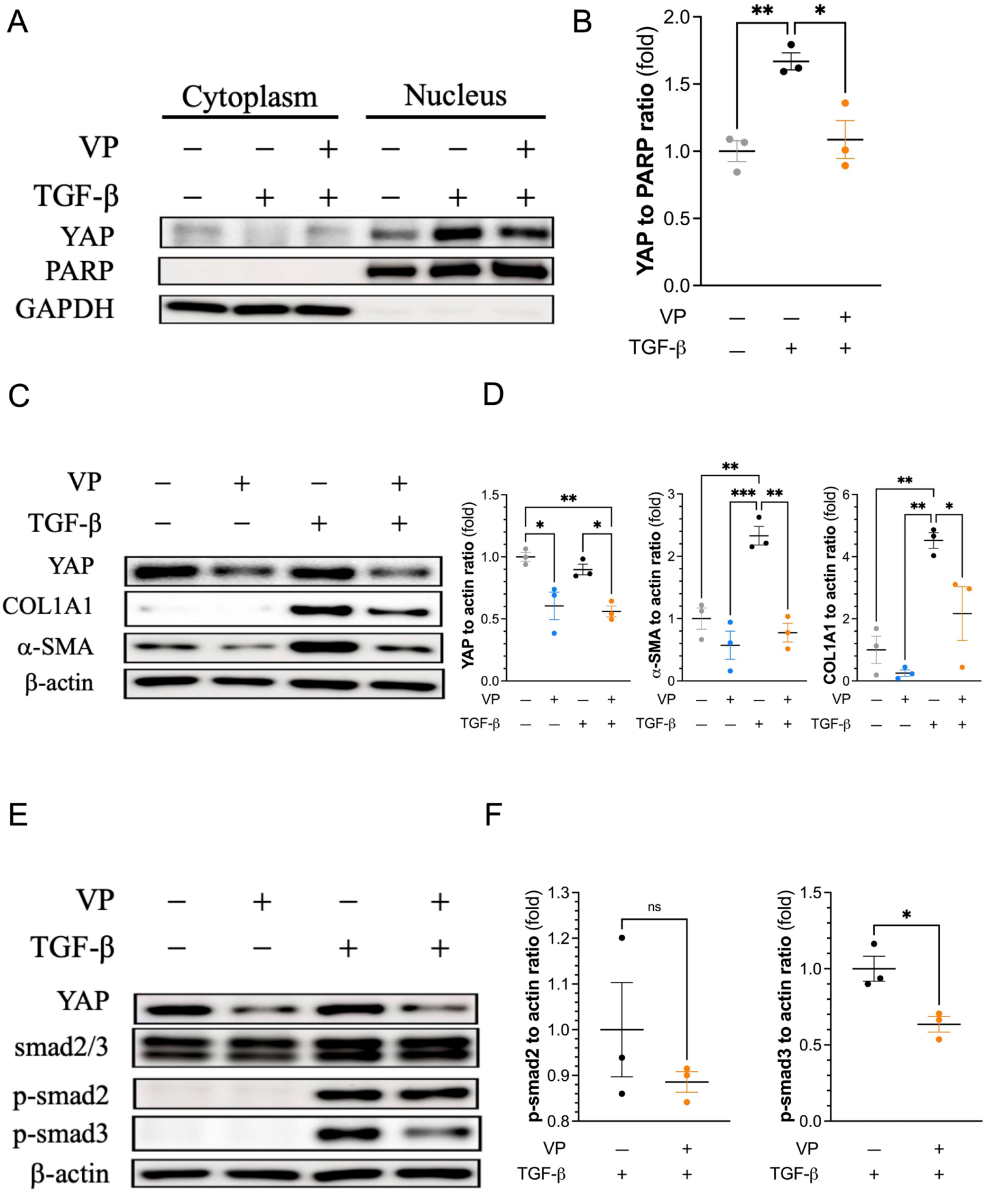
Verteporfin inhibits YAP and fibroblast‐to‐myofibroblast transition. TGF‐β increased YAP nuclear translocation, and verteporfin suppressed the nuclear translocation of YAP in 3T3 cells (A, representative western blots; B, quantitative densitometric analysis of the YAP‐to‐nuclear marker ratio). Verteporfin suppressed total cellular YAP and inhibited the TGF‐β‐induced expression of α‐SMA and collage 1A1 (C, representative western blots; D, quantitative densitometric analysis). Verteporfin inhibited the TGF‐β‐induced phosphorylation of smad3 (E, representative western blots; F, quantitative densitometric analysis). ns, not statistically significant, *p < 0.05, **p < 0.01, ***p < 0.001. One‐way ANOVA followed by Tukey's post hoc test for (B and D), and unpaired Student's *t* test for (F). *n* = 3 for each group. α‐SMA, alpha‐smooth muscle actin; COL1A1, collagen 1A1; CTGF, connective tissue growth factor; GAPDH, glyceraldehyde‐3‐phosphate dehydrogenase; PARP, poly(ADP‐ribose) polymerase; Smad, mothers against decapentaplegic; TGF‐β, transforming growth factor beta; VP, verteporfin; YAP, yes‐associated protein.

We used the YAP inhibitor verteporfin to reduce the expression of YAP in 3T3 cells [18]. Figure 3A,B shows that verteporfin inhibited YAP expression at baseline and after TGF‐β treatment. In addition, verteporfin inhibited FMT by suppressing the expression of α‐SMA and collagen 1A1 in 3T3 cells treated with TGF‐β (Figure 3C,D). Like YAP knockdown, verteporfin tended to suppress the expression of α‐SMA and collagen 1A1 in fibroblasts without TGF‐β (Figure 3C,D). Moreover, verteporfin decreased the phosphorylation of smad3, which was triggered by TGF‐β (Figure 3E,F). Furthermore, verteporfin inhibited FMT in primary mouse fibroblasts (Figure S2B).

### 3.4 Simultaneous Activation of YAP and *Gli1* in Peritoneal Fibrosis

**FIGURE 4:**
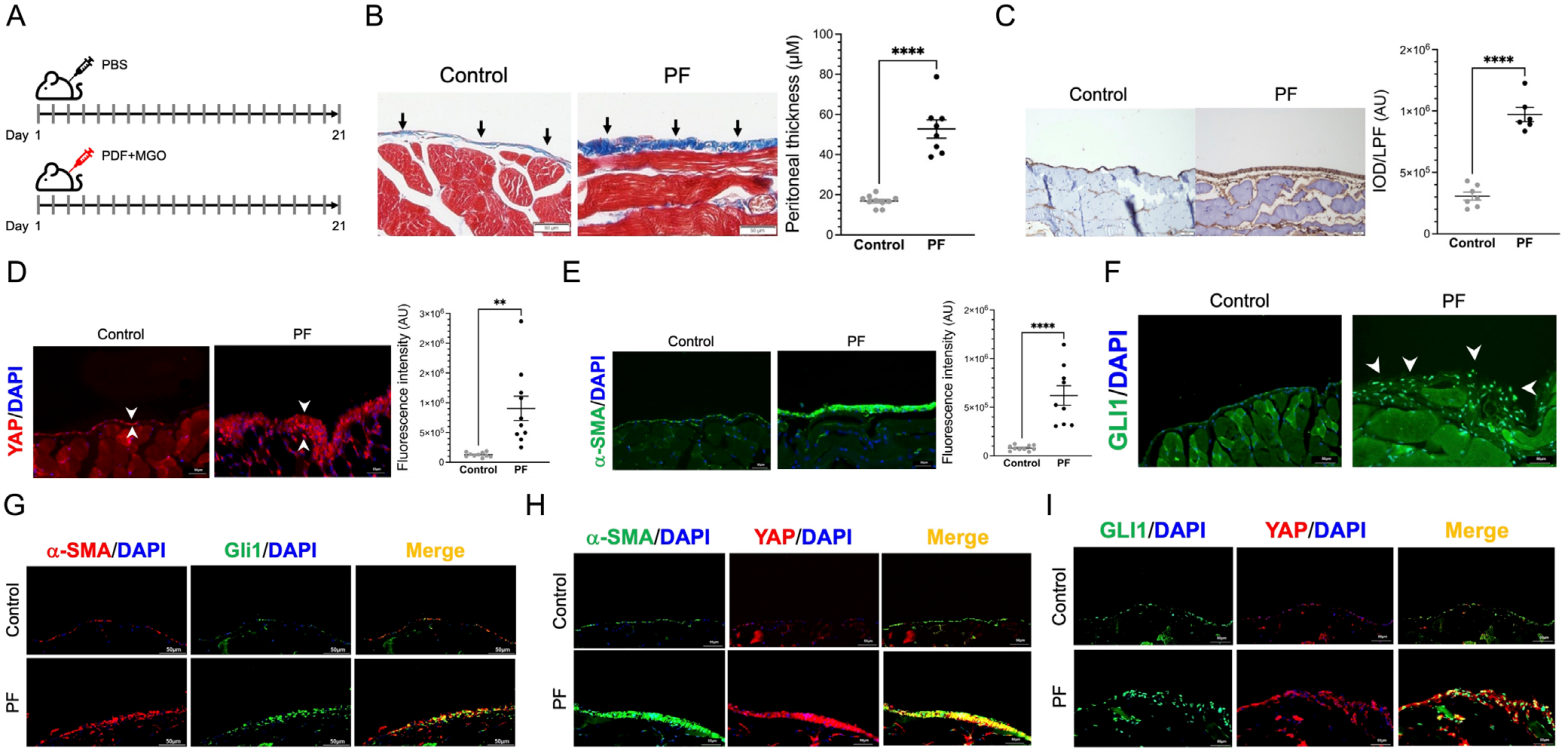
Activation of YAP and *Gli1* during peritoneal dialysis fluid‐induced peritoneal fibrosis. (A) The protocol for inducing peritoneal fibrosis in the mouse model. The mice were intraperitoneally injected with peritoneal dialysis fluid containing 4.25% glucose and 40 mM methylglyoxal daily for 21 days to induce peritoneal fibrosis. Control mice were injected with the same volume of phosphate‐buffered saline. *n* = 6 to 10 for each group. (B) Masson's trichrome staining revealed peritoneal dialysis fluid‐induced peritoneal fibrosis (shown in blue) and increased peritoneal thickness (quantitative analysis shown in the right panel). Scale bar, 50 μm. (C) Increased collagen 1 accumulation was observed in the peritoneum in mice with peritoneal fibrosis (left and middle panels, representative images; right panel, quantitative analysis; scale bar, 50 μm). (D) Peritoneal YAP expression in control mice was low (between white arrowheads, left panel). After the induction of peritoneal fibrosis, the expression of YAP in the peritoneum increased (between white arrowheads, middle panel). The right panel shows the result of the quantitative analysis. Scale bar, 50 μm. (E) The expression of α‐SMA in the peritoneum in control and peritoneal fibrosis mice (left and middle panels, representative images; right panel, quantitative analysis). Scale bar, 50 μm. (F) *Gli1*‐expressing cells in the peritoneum were significantly increased in mice with peritoneal fibrosis. Scale bar, 50 μm. (G) Confocal microscopy showed that activated myofibroblasts expressing α‐SMA also expressed *Gli1* in the fibrotic peritoneum (yellow fluorescence). Scale bar, 50 μm. (H) Confocal microscopy showed that the increases in YAP and α‐SMA largely overlapped (yellow fluorescence) in the peritoneum. Scale bar, 50 μm. (I) YAP upregulation partially colocalized (yellow fluorescence) with *Gli1* ^+^ cells. Scale bar, 50 μm. **p < 0.01, ****p < 0.0001. Unpaired Student's *t* test for (B–E). *n* = 6–10 for each group. α‐SMA, alpha‐smooth muscle Actin; AU, arbitrary unit; *Gli1*, glioma‐associated oncogene 1; IOD, integrated optic density; LPF, low power field; MGO, methylglyoxal; PBS, phosphate buffered saline; PDF, peritoneal dialysis fluid; PF, peritoneal fibrosis; TGF‐β, transforming growth factor beta; YAP, yes‐associated protein.

Intraperitoneal administration of PDF containing 4.25% glucose and methylglyoxal for 21 days was used to induce peritoneal fibrosis (Figure 4A). Compared with those in mice treated with PBS, peritoneal thickness and the accumulation of collagen 1 were significantly increased in mice treated with intraperitoneal PDF for 21 days (Figure 4B,C, and Figure S3). The expression of YAP and α‐SMA was upregulated in the peritoneum after the induction of peritoneal fibrosis (Figure 4D,E, and Figure S3). In addition, the population of *Gli1* ^+^ cells in the peritoneum was increased by the induction of peritoneal fibrosis (Figure 4F). Confocal immunofluorescence microscopy revealed that *Gli1* colocalized with activated myofibroblasts expressing α‐SMA after the induction of peritoneal fibrosis (Figure 4G). Confocal microscopy also revealed the colocalization of α‐SMA and YAP, which were upregulated, in the fibrotic peritoneum (Figure 4H). Moreover, YAP was partially expressed in peritoneal *Gli1*‐expressing cells in mice with peritoneal fibrosis (Figure 4I). Furthermore, the expression of downstream CTGF and the phosphorylation of smad2 and smad3 were increased in the peritoneum of mice with peritoneal fibrosis (Figure S3).

### 3.5 Conditional Knockout of YAP in *Gli1* ^*+*^ Cells Attenuates Peritoneal Fibrosis

**FIGURE 5:**
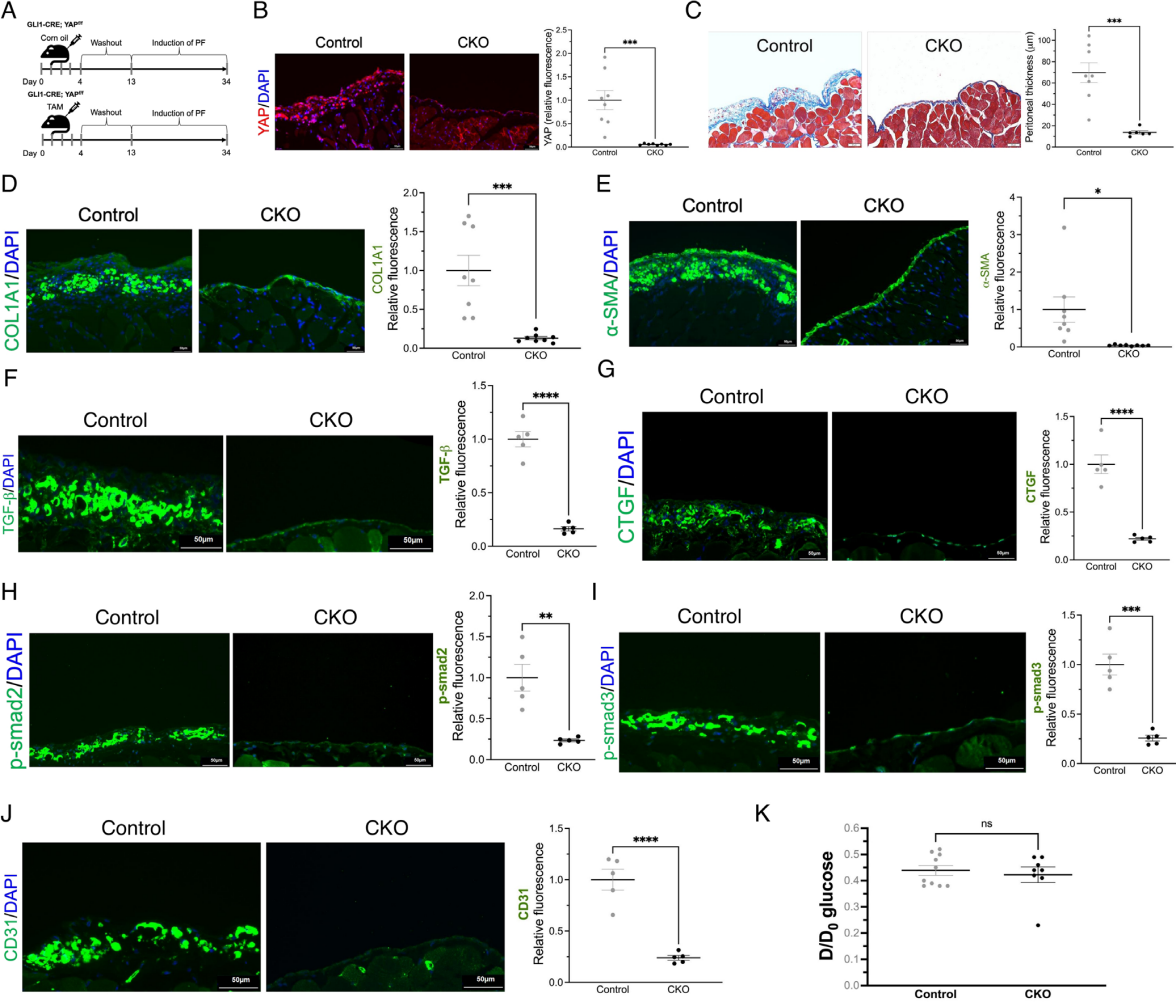
Knockout of YAP in *Gli1*‐expressing cells attenuates peritoneal fibrosis. (A) The protocol for generating YAP conditional knockout mice. *YAP* ^*f/f*^;*Gli1*‐CreERT2^+/−^ mice were intraperitoneally injected with tamoxifen or corn oil in the YAP conditional knockout (CKO) or control groups, respectively. *n* = 8 for each group. (B) YAP CKO silenced the expression of YAP (red fluorescence) in the peritoneum (middle panel) compared to that in the control group (left panel). YAP was expressed in the rectus abdominis muscle (both panels). Scale bar, 50 μm. Quantitative analysis of YAP expression, as measured by immunofluorescence (right panel). (C) Peritoneal thickness (shown in blue) was significantly reduced in the CKO group compared with the control group. Scale bar, 50 μm. (D) Representative images of peritoneal collagen 1A1 accumulation in the control and CKO groups (left and middle panels). Scale bar, 50 μm. The right panel shows the result of the quantitative analysis. (E) Representative images of peritoneal α‐SMA expression in the control and CKO groups (left and middle panels). Scale bar, 50 μm. The right panel shows quantitative analysis of peritoneal α‐SMA expression in the control and CKO groups. (F–I) Peritoneal expressions of TGF‐β (F), CTGF (G), phosphorylated smad2 (H), and phosphorylated smad3 (I) were suppressed in the YAP CKO group (J) CD31 staining slowed lower blood vessel density in the YAP CKO group. Scale bar, 50 μm. (K) D/D_0_ glucose did not significantly change. ns, not significant; *p < 0.05, **p < 0.01, ***p < 0.001, ****p < 0.0001. Unpaired Student's *t* test for (B–K). *n* = 5 to 9 for each group. α‐SMA, alpha‐smooth muscle actin; CD, cluster of differentiation; CKO, conditional knockout; COL1A1, collagen 1A1; CTGF, connective tissue growth factor; D/D_0_ glucose, the ratio of dialysate glucose concentration at the end of the test to the start; DAPI, 4′, 6‐diamidino‐2‐phenylindole; *Gli1*, glioma‐associated oncogene 1; PF, peritoneal fibrosis; Smad, mothers against decapentaplegic; TAM, tamoxifen; TGF‐β, transforming growth factor beta; YAP, yes‐associated protein.

^*f/f*^ *YAP; Gli1*‐CreERT2^+/−^ mice were intraperitoneally administered tamoxifen or the same volume of corn oil before the induction of peritoneal fibrosis (Figure 5A). YAP expression in the peritoneum was substantially increased during peritoneal fibrosis (Figure 5B, left panel) but was decreased by YAP CKO (Figure 5B, middle and right panels). *Gli1* expression in the peritoneum was also suppressed by the CKO (Figure S4). YAP CKO significantly ameliorated the development of PDF‐induced peritoneal fibrosis (Figure 5C). The accumulation of collagen 1 and activation of myofibroblasts, as shown by an increase in α‐SMA, were significantly reduced in the YAP CKO group compared to the control group (Figure 5D,E, and Figure S4). Moreover, the expression of TGF‐β, CTGF, and phosphorylated smad2/3 was suppressed in the YAP CKO group (Figure 5F–I and Figure S4). To evaluate angiogenesis in the peritoneal membrane, CD31 staining was performed and revealed significantly lower blood vessel density in the YAP CKO group (Figure 5J). The peritoneal solute transfer rate has been reported to correlate with angiogenesis [17]. However, the difference in the peritoneal solute transfer rate measured by the ratio of dialysate glucose concentration at the end of the test to the start (D/D_0_ glucose) was insignificant between the control and YAP CKO groups (Figure 5K).

### 3.6 Verteporfin Attenuates In Vivo Peritoneal Fibrosis

**FIGURE 6:**
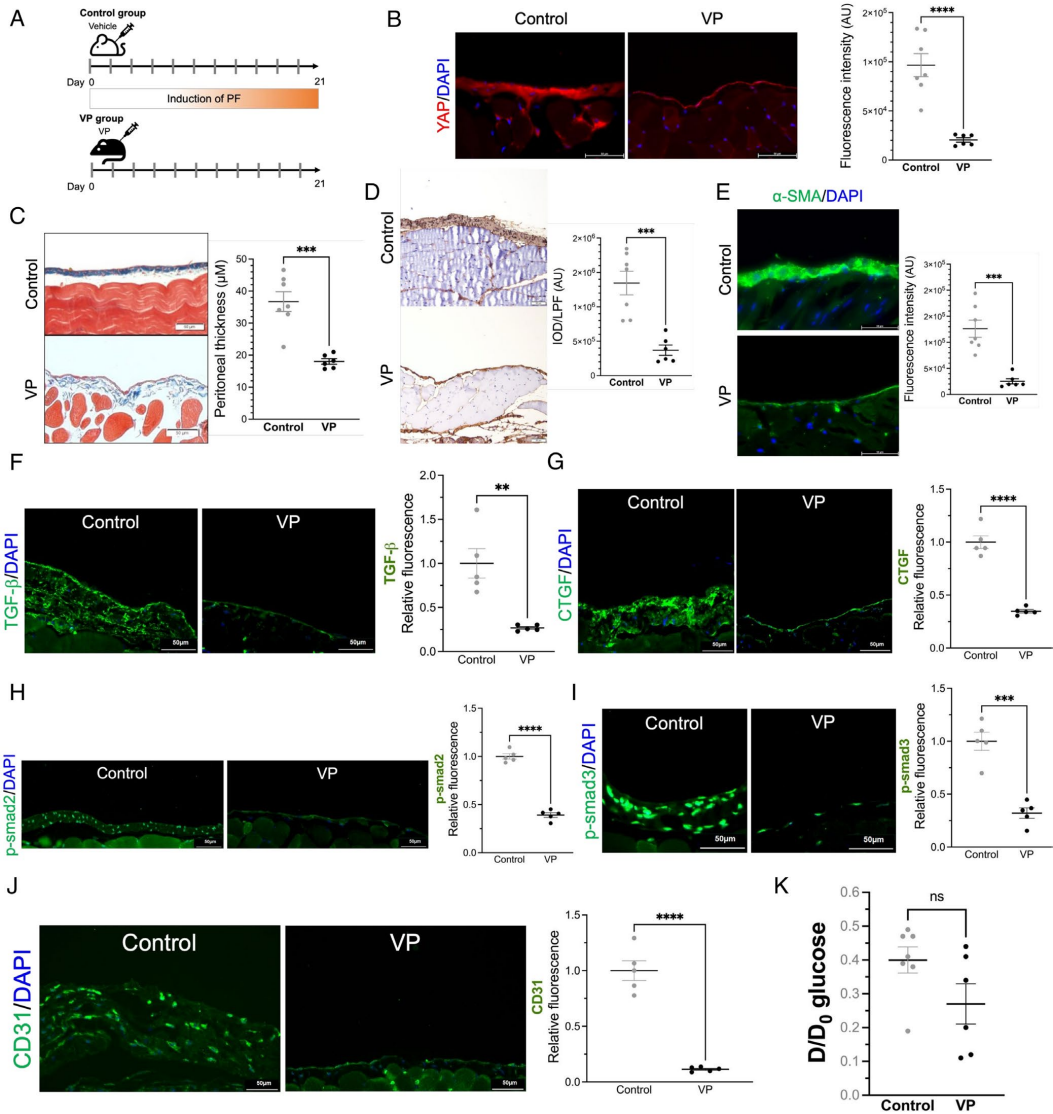
The effect of intraperitoneal verteporfin on peritoneal fibrosis. (A) The protocol of verteporfin (VP) treatment and induction of peritoneal fibrosis in the mouse model. *n* = 6–7 for each group. (B) YAP expression in the peritoneum was significantly lower in the VP group than in the control group (left and middle panels, representative images; right panel, quantitative analysis of YAP fluorescence intensity). Scale bar, 50 μm. (C) Peritoneal fibrosis was ameliorated in the VP group compared with the control group (representative images; left‐upper and ‐lower panels). The right panel shows the quantitative analysis result comparing the peritoneal thickness between the VP and control groups. Scale bar, 50 μm. (D) Less collagen 1A1 accumulated in the peritoneum in the VP group than in the control group (representative images; left‐upper and ‐lower panels). Quantitative analysis results comparing the amount of collage 1A1 in the peritoneum between the VP and control groups (right panel). Scale bar, 50 μm. (E) α‐SMA expression in the peritoneum was substantially suppressed in the VP group compared with the control group (representative images; left‐upper and ‐lower panels). The quantitative analysis result is shown in the right panel. Scale bar, 50 μm. (F–J) Peritoneal expressions of TGF‐β (F), CTGF (G), phosphorylated smad2 (H), phosphorylated smad3 (I), and CD31 (J) were inhibited by verteporfin treatment. Scale bar, 50 μm. (K) D/D_0_ glucose between the control and VP groups did not significantly differ. ns, not significant; **p < 0.01, ***p < 0.001, ****p < 0.0001. Unpaired Student's *t* test for (B–J). *n* = 5–7 for each group. α‐SMA, alpha‐smooth muscle actin; AU, arbitrary unit; CD, cluster of differentiation; COL1A1, collagen 1A1; CTGF, connective tissue growth factor; D/D_0_ glucose, the ratio of dialysate glucose concentration at the end of the test to the start; DAPI, 4′, 6‐diamidino‐2‐phenylindole; IOD, integrated optic density; LPF, low power field; PF, peritoneal fibrosis; Smad, mothers against decapentaplegic; TGF‐β, transforming growth factor beta; VP, verteporfin; YAP, yes‐associated protein.

Verteporfin was intraperitoneally administered every other day for 21 days to inhibit YAP expression in the peritoneum (Figure 6A). YAP expression in the peritoneum was inhibited in mice treated with verteporfin compared with those in the control group (Figure 6B). In addition, verteporfin treatment attenuated peritoneal thickening (Figure 6C) and the production of collagen 1A1 in the peritoneum (Figure 6D), which were exaggerated in the control group. The PDF‐induced increase in α‐SMA expression was also ameliorated by verteporfin treatment (Figure 6E). Moreover, verteporfin treatment suppressed the expression of TGF‐β, CTGF, phosphorylated smad2/3, and CD31 (Figure 6F–J). Like YAP CKO, the D/D_0_ glucose between the control and verteporfin‐treated mice did not significantly differ (Figure 6K).

## 4 Discussion

**FIGURE 7:**
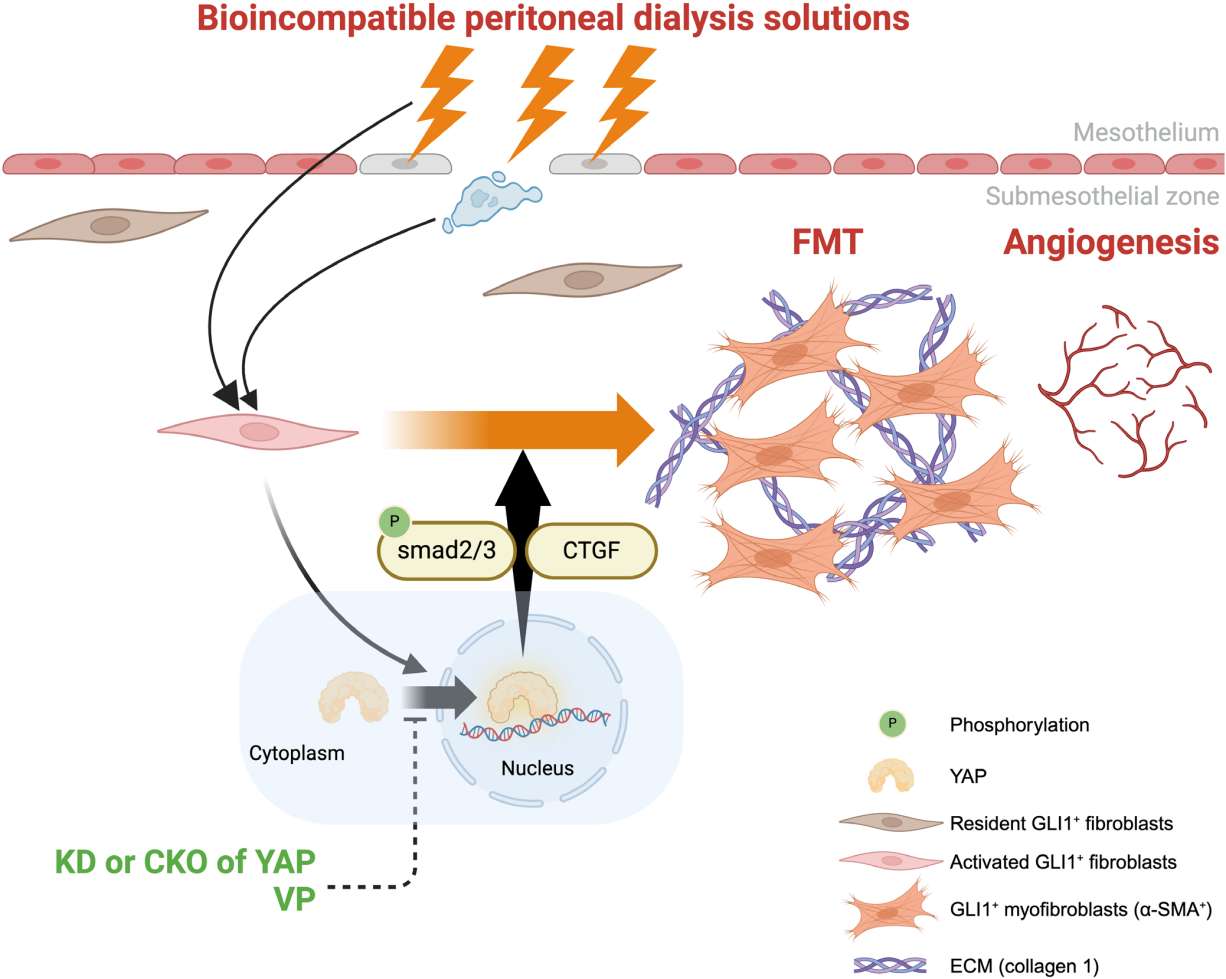
The schematic diagram of the study. Exposure to peritoneal dialysis fluid containing high glucose concentrations and glucose degradation products upregulates YAP expression and triggers YAP nuclear translocation. The increase in YAP expression in the peritoneum can promote the activation of myofibroblasts (FMT) and result in peritoneal fibrosis and angiogenesis with increased expression levels of α‐SMA, CTGF, phosphorylated smad2/3, and extracellular matrix (collagen 1). Knocking out YAP in *Gli1*‐expressing cells or verteporfin treatment depletes YAP in the peritoneum and attenuates peritoneal fibrosis and angiogenesis. α‐SMA, alpha‐smooth muscle actin; CKO, conditional knockout; CTGF, connective tissue growth factor; FMT, fibroblast‐to‐myofibroblast transition; *Gli1*, glioma‐associated oncogene 1; KD, knockdown; smad, mothers against decapentaplegic; VP, verteporfin; YAP, yes‐associated protein.

The current study demonstrates the essential role of YAP in PD‐induced peritoneal fibrosis (Figure 7). PDF containing glucose at supraphysiologic concentrations and glucose degradation products such as methylglyoxal, as well as an acidic pH, promoted YAP expression and nuclear translocation, activated the FMT, increased the population of myofibroblasts and the levels of myofibroblast‐produced collagen 1, and led to peritoneal fibrosis. Knockdown of YAP by siRNA and verteporfin treatment inhibited the FMT in fibroblasts. Moreover, YAP CKO and verteporfin treatment ameliorated PDF‐induced peritoneal fibrosis and angiogenesis.

The Hippo pathway is a highly evolutionarily conserved signalling pathway that was first reported to control the growth of organs [19]. Studies have shown the effects of the Hippo pathway on tissue homeostasis and tumorigenesis [19]. YAP and TAZ are key downstream effectors of the Hippo pathway and mediate epithelial‐to‐mesenchymal transition and fibrosis in the kidney [13, 20]. Liang reported that the transformation of fibroblasts to myofibroblasts depended on YAP activation [13]. A recent study further demonstrated that YAP and TAZ activation in injury‐induced myofibroblasts was a critical driver of organ fibrosis [21]. A previous study revealed the profibrotic effect of caveolin‐1 and YAP on the mesothelial‐to‐mesenchymal transition (MMT) [14]. However, they did not show direct evidence that YAP/TAZ independently promoted MMT in vitro or peritoneal fibrosis in vivo. In the present study, we demonstrated that YAP is essential for FMT in vitro and PDF‐induced peritoneal fibrosis in vivo.

*Gli1*‐expressing cells have been shown to differentiate into myofibroblasts during renal fibrosis [22]. Our study revealed that the population of *Gli1* ^+^ cells was expanded in the peritoneum, especially in the submesothelial zone. After the induction of peritoneal fibrosis, these *Gli1* ^+^ cells expressed α‐SMA, suggesting that these cells were activated by PDF and transdifferentiated into myofibroblasts. In addition, YAP was upregulated in these activated *Gli1* ^+^ myofibroblasts. Moreover, depleting YAP expression in *Gli1*‐expressing cells or verteporfin treatment reduced the severity of the peritoneal fibrosis induced by PDF. Our results demonstrate the essential role of YAP in activated *Gli1* ^+^ myofibroblasts and the development of peritoneal fibrosis.

The Hippo pathway promotes the cytoplasmic localisation of YAP/TAZ, which sequesters Smad proteins, thereby inhibiting the TGF‐β signalling pathway [23]. TGF‐β was reported to induce the formation of YAP/TAZ‐smad2/3 complexes in the nuclei of HaCaT cells [24]. Liu et al. [25] reported that smooth muscle cell‐specific knockdown of YAP ameliorated high‐fat/high‐sucrose diet‐induced arterial stiffness and activation of the TGF‐β‐smad2/3 signalling pathway. In addition, Szeto et al. reported that TGF‐β induced renal fibrosis in a YAP/TAZ‐smad2/3‐dependent manner [26]. Our study showed that TGF‐β promoted FMT and the activation of smad2/3 in a YAP‐dependent manner. YAP CKO also reduced the expression and phosphorylation of smad2/3 in the peritoneal membrane. Thus, we suggest that YAP plays an essential role in canonical TGF‐β‐smad2/3 signalling through the TGF‐β‐YAP‐smad2/3 pathway during the development of peritoneal fibrosis. Further mechanistic research is needed to confirm our results.

CTGF is a member of the CCN family, which is extracellular matrix‐associated proteins [27]. CTGF can regulate cell proliferation, differentiation, and adhesion. Previous studies have shown that CTGF is involved in the development of peritoneal fibrosis and that depleting CTGF ameliorates peritoneal fibrosis and inhibits inflammation and angiogenesis [28, 29, 30]. CTGF is also a downstream target gene of YAP [10]. Our findings demonstrate that YAP regulates α‐SMA and CTGF expression in fibroblasts. Yu et al. demonstrated that YAP is essential for activating actin alpha 2 (*ACTA2*, encoding α‐SMA) and cellular communication network factor 2 (*CCN2*, encoding CTGF), using multiple siRNA constructs and cell types to validate specificity [31]. The mechanistic interaction between YAP and these profibrotic genes was proposed based on the proximity between the binding sites of transcriptional enhanced associate domain proteins and the promoter regions of *ACTA2* and *CCN2* according to previous chromatin immunoprecipitation‐sequencing (ChIP‐seq) studies [31]. Additionally, Hu et al. confirmed through direct ChIP assays that YAP binds to the CTGF promoter, activating its expression in neonatal cardiac fibroblasts [32]. In vivo, YAP knockdown decreases profibrotic TGF‐β and CTGF levels in the kidney after renal IRI [33]. Hu et al. also showed that YAP inhibition alleviated myocardial fibrosis by downregulating collagen I, collagen III, and CTGF in an in vivo model of diabetic cardiomyopathy [32]. Here, we showed that YAP knockdown inhibited CTGF expression during FMT and peritoneal fibrosis. Moreover, YAP conditional knockout or verteporfin treatment ameliorated angiogenesis induced by PDFs, as YAP has been shown to regulate angiogenesis [34]. Our results are consistent with previous findings and fill the knowledge gap in the field of peritoneal fibrosis.

There are limitations in the current study. First, we only knocked out YAP in *Gli1*‐expressing cells. We only examined the effect of depleting YAP in *Gli1* ^+^ myofibroblasts. YAP may also participate in fibrogenesis in other cell types in the peritoneum, and further research is needed to clarify this issue. Second, tamoxifen itself can affect fibrosis. The use of tamoxifen to induce CKO may confound the results of a fibrosis study using female mice [35]. Thus, the experiments in the current study were performed with male mice. Tamoxifen reportedly ameliorated the fibrotic response in the peritoneal membrane. It inhibited peritoneal smad3 expression in uremic rats with peritoneal fibrosis [36]. To compare the effect on peritoneal fibrosis in mice of the same genetic background, tamoxifen or corn oil was administered for 5 consecutive days to CKO or control mice. Our study included a 9‐day washout period before the induction of peritoneal fibrosis. However, the 9‐day washout period for tamoxifen might not be long enough. This potential bias was tested, and the results showed that YAP CKO significantly suppressed peritoneal fibrosis compared with the controls, which were wild‐type mice injected intraperitoneally with tamoxifen (Figure S5). Furthermore, the Cre‐loxP system might affect fibrosis. However, a recent study reported that they did not find different collagen deposits and fibrotic responses between the presence of Cre recombinase or not [37], indicating that Cre recombinase per se may not affect fibrosis. The third limitation of our study is the absence of direct analysis of human peritoneal biopsy samples, particularly immunostaining for *Gli1* and YAP from specimens obtained at the time of peritoneal catheter removal. While previous studies have provided evidence supporting the roles of YAP in peritoneal fibrosis and mesothelial‐to‐mesenchymal transition, including data from human peritoneal biopsies [14], we were unable to replicate these findings due to the unavailability of clinical specimens. Similarly, the potential involvement of *Gli1* in peritoneal fibrosis, which has been linked to Hedgehog signalling and fibroblast activation in other tissues, warrants further exploration in this context. Future studies involving a larger set of human biopsy samples with specific immunostaining for *Gli1* and YAP are needed to confirm and extend these observations and strengthen the translational relevance of our findings. Finally, it is unclear whether the results obtained from mice and in vitro studies could be applied to humans. Further translational studies are needed to clarify the therapeutic effects of targeting *Gli1*‐specific YAP expression on peritoneal fibrosis or EPS.

## 5 Conclusions

In summary, YAP plays an essential role in the pathogenesis of peritoneal fibrosis. YAP promotes fibroblast transdifferentiation to myofibroblasts. Depleting YAP in *Gli1* ^+^ cells inhibits FMT and the development of PDF‐induced peritoneal fibrosis and angiogenesis. In addition, verteporfin suppresses peritoneal YAP expression and inhibits FMT and peritoneal fibrosis and angiogenesis. Our results suggest that YAP is a novel therapeutic target and that verteporfin may be a candidate for preventing or treating peritoneal fibrosis in patients undergoing long‐term PD.

## Author Contributions

**Chia‐Lin Wu:** conceptualization (equal), formal analysis (equal), funding acquisition (lead), investigation (lead), methodology (equal), project administration (lead), resources (lead), supervision (equal), validation (equal), visualization (equal), writing – original draft (lead), writing – review and editing (equal). **Jhih‐Wen Hsu:** data curation (equal), formal analysis (equal), methodology (equal), validation (equal). **Ya‐Chi Chan:** data curation (equal), formal analysis (equal), methodology (equal), software (equal), validation (equal), visualization (equal). **Jenn‐Yah Yu:** methodology (equal), resources (equal), writing – review and editing (equal). **Yi‐Liang Tsai:** formal analysis (equal), software (equal), writing – review and editing (equal). **Der‐Cherng Tarng:** conceptualization (equal), project administration (equal), supervision (equal), validation (equal), writing – original draft (equal), writing – review and editing (equal).

## Ethics Statement

All animal experiments were conducted according to institutional ethics and safety guidelines, and all animal use protocols were approved by the Institutional Animal Care and Use Committee of Changhua Christian Hospital (Changhua, Taiwan).

## Conflicts of Interest

The authors declare no conflicts of interest.

## Supporting information


**Figure S1.** Genotypes of mice for conditional knockout experiments.
**Figure S2.** Suppressing YAP prohibited fibroblast‐to‐myofibroblast transition (FMT) in primary mouse fibroblasts.
**Figure S3.** Peritoneal protein expressions in the peritoneum between mice with intraperitoneal phosphate‐buffered saline (control) or peritoneal dialysis fluid (PF).
**Figure S4.** Protein expressions in the peritoneum between control mice (control) YAP conditional knockout mice (CKO).
**Figure S5.** YAP conditional knockout significantly ameliorated peritoneal fibrosis compared to wild‐type mice treated with tamoxifen.

## Data Availability

All data used or analysed in this study were included in the main text and Supporting Information of this article. All relevant raw data will be freely available to any researcher wishing to use them for noncommercial purposes. Further enquiries can be directed to the corresponding authors.
