# Supplementary material for: Depleting Yes‐Associated Protein in Gli1‐Expressing Cells Attenuates Peritoneal Dialysis‐Induced Peritoneal Fibrosis
Source: J Cell Mol Med. 2025 Mar 25;29(6):e70516. doi: 10.1111/jcmm.70516 (PMC11936727; doi:10.1111/jcmm.70516)
Supplement: Supplementary file 1 — Figure S1. Genotypes of mice for conditional knockout experiments. Figure S2. Suppressing YAP prohibited fibroblast‐to‐myofibroblast transition (FMT) in primary mouse fibroblasts. Figure S3. Peritoneal protein expressions in the peritoneum between mice with intraperitoneal phosphate‐buffered saline (control) or peritoneal dialysis fluid (PF). Figure S4. Protein expressions in the peritoneum between control mice (control) YAP conditional knockout mice (CKO). Figure S5. YAP conditional knockout significantly ameliorated peritoneal fibrosis compared to wild‐type mice treated with tamoxifen. [file JCMM-29-e70516-s001.pdf]

Depleting yes-associated protein in *Gli1*-expressing cells attenuates peritoneal dialysis-induced peritoneal fibrosis

Short running title: YAP Knockout and Peritoneal Fibrosis

Chia-Lin Wu[1,2,3,4*], Jhih-Wen Hsu[4], Ya-Chi Chan[4], Jenn-Yah Yu[5], Yi-Liang Tsai[4], and Der-Cherng Tarng[6,7*]

[1] Department of Post-Baccalaureate Medicine, College of Medicine, National Chung Hsing University, Taichung, Taiwan

[2] School of Medicine, Chung Shan Medical University, Taichung, Taiwan

[3] Division of Nephrology, Department of Internal Medicine, Changhua Christian Hospital, Changhua, Taiwan

[4] Renal Medicine Laboratory, Changhua Christian Hospital, Changhua, Taiwan

[5] Department of Life Sciences and Institute of Genome Sciences, National Yang Ming Chiao Tung University, Taipei, Taiwan

[6] Department and Institute of Physiology, National Yang Ming Chiao Tung University, Taipei, Taiwan

[7] Division of Nephrology, Department of Medicine, Taipei Veterans General Hospital, Taipei, Taiwan

***Correspondence (*):** Dr. Der-Cherng Tarng, Department and Institute of Physiology, National Yang Ming Chiao Tung University, and Division of Nephrology, Department of Medicine, Taipei Veterans General Hospital, No. 201, Section 2, Shih-Pai Road, Taipei 11217, Taiwan. E-mail: dctarng@vghtpe.gov.tw and Dr. Chia-Lin Wu, Division of Nephrology, Department of Internal Medicine, Changhua Christian Hospital, No. 135, Nan-Siau Street, Changhua 500, Taiwan. E-mail: 143843@cch.org.tw

Supplementary Methods

Supplementary References

Supplementary Figure S1. Genotypes of mice for conditional knockout experiments.

Supplementary Figure S2. Suppressing YAP prohibited fibroblast-to-myofibroblast transition (FMT) in primary mouse fibroblasts.

Supplementary Figure S3. Peritoneal protein expressions in the peritoneum between mice with intraperitoneal phosphate-buffered saline (control) or peritoneal dialysis fluid (PF).

Supplementary Figure S4. Protein expressions in the peritoneum between control mice (control) YAP conditional knockout mice (CKO).

Supplementary Figure S5. YAP conditional knockout significantly ameliorated peritoneal fibrosis compared to wild-type mice treated with tamoxifen.

Supplementary Methods

Primary mouse fibroblasts

Primary fibroblasts were isolated as previously described with some modifications.[1, 2] In brief, ear and tail tissues were detached from mice, disinfected with 70% ethanol, minced into small pieces, and incubated for 90 min at 37°C in the mixture solution of collagenase D (catalog no. 11088866001, Roche Diagnostics) and pronase protease (catalog no. 53702, Merck Millipore). Digested tissues were placed in the 70-μm cell strainer and were ground using a 10-ml syringe plunger for more than 5 min. Cells were then suspended in Roswell Park Memorial Institute 1640 medium (catalog no. SH30027.01, HyClone) supplemented with 10% fetal calf serum (catalog no. SV30160.03, HyClone), 1% penicillin-streptomycin (catalog no. SV30010, HyClone), 100 μM asparagine (catalog no. A4159, Sigma-Aldrich), 2 mM glutamine (catalog no. G8540, Sigma-Aldrich), and 50 μM 2-mercaptoethanol (catalog no. M3148, Sigma-Aldrich). 10 μl of amphotericin B solution (250 μg/ml, catalog no. A2492, Sigma-Aldrich) were added to the culture and cells were incubated at 37 °C in a humidified 5% CO_2 incubator. When 70% confluence was achieved, the cells were subdivided into culture dishes (2 x 10^5 cells/each dish). Primary cells were treated with YAP siRNA (1 μg, SASI Mm01_00022141/YAP1, Sigma; sequence: 5´-CCAAUAGUUCCGAUCCCUU-3´), or 500 nM verteporfin (catalog no. 17334, Cayman) with 10 ng/ml TGF-β (catalog no. 763102, BioLegend) for 24 h.

Peritoneal Equilibration Test (PET)

Peritoneal solute transport was evaluated using a modified method from a previous study.[3] Wild-type, conditional knockout, or verteporfin treatment mice were treated with 4.25% glucose peritoneal dialysis (PD) solution (Dianeal, Baxter Healthcare)

daily for 3 weeks. Then, PET was performed using 2 mL of 2.5% glucose PD solution (Dianeal, Baxter Healthcare). Solutions were collected after 30 minutes of retention. Dialysate glucose levels were measured using the enzymatic method. Glucose concentration was analyzed in the PD solution at 0 and 30 min of dwell time. The glucose concentration ratio at 30 and 0 min (D/D_0) was obtained to evaluate peritoneal solute transport.

Supplementary References

1. Khan M, Gasser S. Generating Primary Fibroblast Cultures from Mouse Ear and Tail Tissues. J Vis Exp. 2016;107:53565.
2. Ishimura T, Ishii A, Yamada H, et al. Matrix metalloproteinase-10 deficiency has protective effects against peritoneal inflammation and fibrosis via transcription factor NFκB pathway inhibition. Kidney Int. 2023;104(5):929-942.
3. Yi-Ting Chen, Hao Hsu, Chi-Chun Lin, et al. Inflammatory macrophages switch to CCL17-expressing phenotype and promote peritoneal fibrosis. J Pathol. 2020;250(1):55-66.

Supplementary Figure S1. Genotypes of mice for conditional knockout experiments.

The genotyping results of mice A, B, and C were *YAP*$^{f/f}$;*Gli1*-CreERT2$^{+/-}$ and D was *YAP*$^{f/-}$;*Gli1*-CreERT2$^{+/-}$.

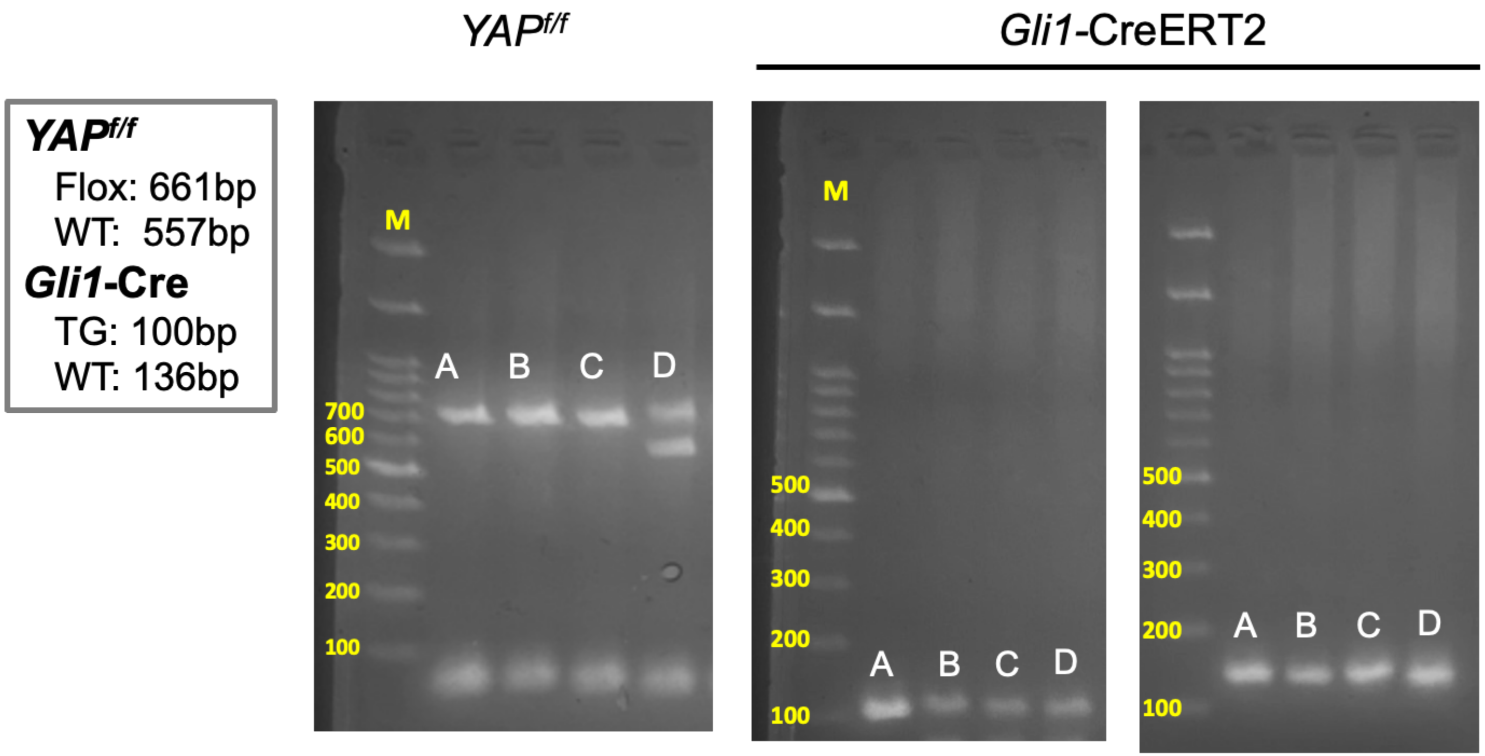

Abbreviations: *f*, gene flanked by loxP sites; TG, transgene; WT, wild type.

Supplementary Figure S2. Suppressing YAP prohibited fibroblast-to-myofibroblast transition (FMT) in primary mouse fibroblasts.

Suppressing YAP expression by small interfering RNA (A) or verteporfin (B) decreased transforming growth factor beta-induced expressions of YAP, alpha-smooth muscle actin, and collagen 1A1 in primary mouse fibroblasts. * p<0.05, ** p<0.01.

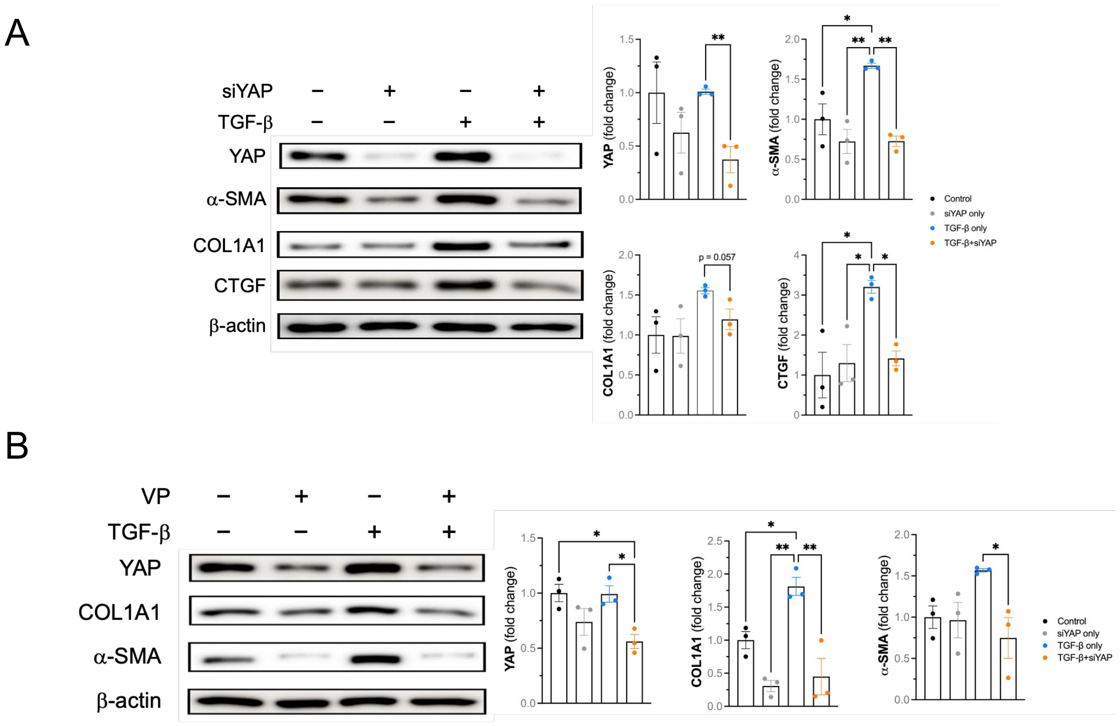

Abbreviations: α-SMA, alpha-smooth muscle actin; COL1A1, collagen 1A1; CTGF, connective tissue growth factor; TGF-β, transforming growth factor beta; YAP, yes-associated protein.

Supplementary Figure S3. Peritoneal protein expressions in the peritoneum between mice treated with intraperitoneal phosphate-buffered saline (control) or peritoneal dialysis fluid (PF).

Western blotting shows that the peritoneum of mice with peritoneal fibrosis contained higher expressions of transforming growth factor beta, yes-associated protein, alpha-smooth muscle actin, collagen 1A1, connective tissue growth factor, and phosphorylated and total smad2/3 than that of control mice.

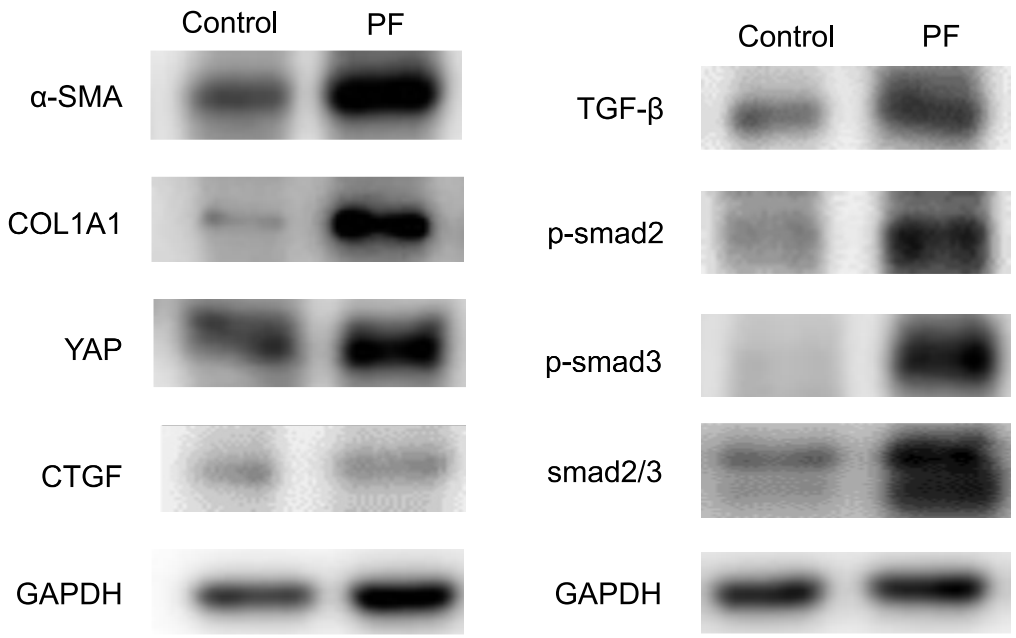

Abbreviations: α-SMA, alpha-smooth muscle actin; COL1A1, collagen 1A1; CTGF, connective tissue growth factor; GAPDH, glyceraldehyde-3-phosphate dehydrogenase; PF, peritoneal fibrosis; smad, mothers against decapentaplegic; TGF-β, transforming growth factor beta; YAP, yes-associated protein.

Supplementary Figure S4. Protein expressions in the peritoneum between control mice (control) and YAP conditional knockout mice (CKO).

Western blotting shows that the peritoneal expressions of *Gli1*, transforming growth factor beta, alpha-smooth muscle actin, collagen 1A1, connective tissue growth factor, and phosphorylated and total smad2/3 were suppressed in CKO mice compared with control mice.

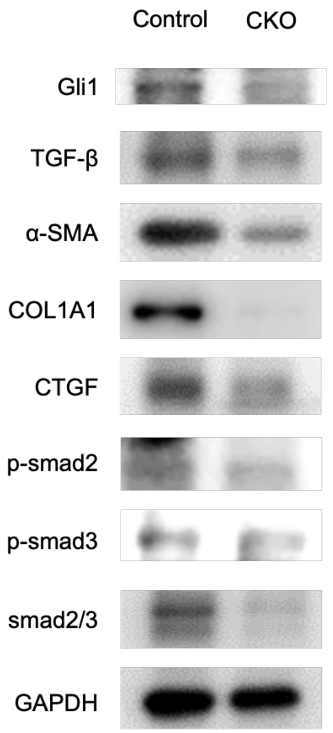

Abbreviations: α-SMA, alpha-smooth muscle actin; COL1A1, collagen 1A1; CTGF, connective tissue growth factor; GAPDH, glyceraldehyde-3-phosphate dehydrogenase; *Gli1*, glioma-associated oncogene homolog 1; smad, mothers against decapentaplegic; TGF-β, transforming growth factor beta; YAP, yes-associated protein.

Supplementary Figure S5. YAP conditional knockout significantly ameliorated peritoneal fibrosis compared to wild-type C57BL/6J mice treated with tamoxifen.

Masson's trichrome stain shows that peritoneal fibrosis (shown in the blue area), measured by the thickness of the peritoneum, was significantly ameliorated in the CKO group compared with the WT+TAM group. Both groups underwent the same protocol of tamoxifen treatment. *** p<0.001. n = 6 for each group.

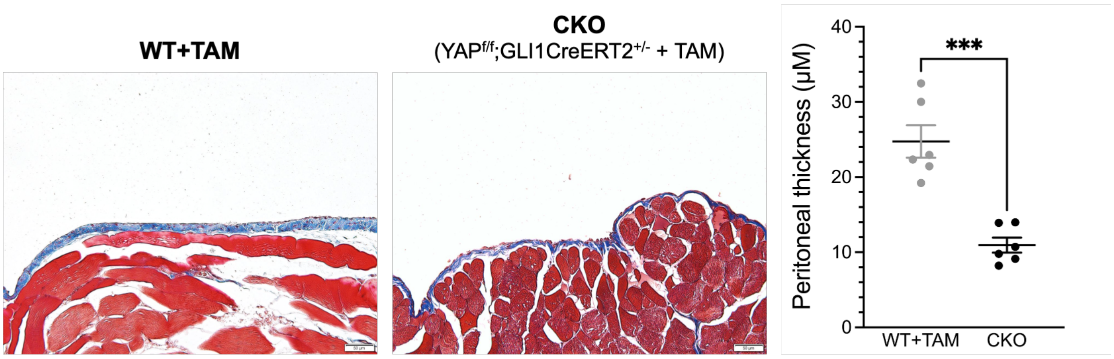

Abbreviations: CKO, conditional knockout; TAM, tamoxifen; WT, wild type.
